# Aberrant phenotypes of transgenic mice expressing dimeric human erythropoietin

**DOI:** 10.1186/1477-7827-10-6

**Published:** 2012-01-27

**Authors:** Seong-Jo Yun, Purevjargal Naidansuren, Bo-Woong Sim, Jong-Ju Park, Cha-Won Park, Tseeleema Nanjidsuren, Myung-Hwa Kang, Sue-Yun Hwang, Jong-Taek Yoon, Kwan-Sik Min

**Affiliations:** 1 Animal Biotechnology, Graduate School of Bio & Information Technology, Institute of Genetic Engineering, Hankyong National University, Ansung 456-749, Korea; 2 Department of Food and Nutrition, Hoseo University, Asan 336-795, Korea

## Abstract

**Background:**

Dimeric human erythropoietin (dHuEPO) peptides are reported to exhibit significantly higher biological activity than the monomeric form of recombinant EPO. The objective of this study was to produce transgenic (tg) mice expressing dHuEPO and to investigate the characteristics of these mice.

**Methods:**

A dHuEPO-expressing vector under the control of the goat beta-casein promoter, which produced a dimer of human EPO molecules linked by a 2-amino acid peptide linker (Asp-Ile), was constructed and injected into 1-cell fertilized embryos by microinjection. Mice were screened using genomic DNA samples obtained from tail biopsies. Blood samples were obtained by heart puncture using heparinized tubes, and hematologic parameters were assessed. Using the microarray analysis tool, we analyzed differences in gene expression in the spleens of tg and control mice.

**Results:**

A high rate of spontaneous abortion or death of the offspring was observed in the recipients of dHuEPO embryos. We obtained 3 founder lines (#4, #11, and #47) of tg mice expressing the *dHuEPO *gene. However, only one founder line showed stable germline integration and transmission, subsequently establishing the only transgenic line (#11). We obtained 2 F1 mice and 3 F2 mice from line #11. The dHuEPO protein could not be obtained because of repeated spontaneous abortions in the tg mice. Tg mice exhibited symptoms such as short lifespan and abnormal blood composition. The red blood cell count, white blood cell count, and hematocrit levels in the tg mice were remarkably higher than those in the control mice. The spleens of the tg mice (F1 and F2 females) were 11- and -21-fold larger than those of the control mice. Microarray analysis revealed 2,672 spleen-derived candidate genes; more genes were downregulated than upregulated (849/764). Reverse transcriptase-polymerase chain reaction (RT-PCR) and quantitative real-time PCR (qRT-PCR) were used for validating the results of the microarray analysis of mRNA expression.

**Conclusions:**

In conclusion, dHuEPO tg mice caused excessive erythrocytosis that led to abnormal blood composition, short lifespan, and abnormal splenomegaly. Further, we identified 2,672 genes associated with splenomegaly by microarray analysis. These results could be useful in the development of dHuEPO-producing tg animals.

## Background

Erythropoietin (EPO), a 30.4-kDa glycoprotein hormone secreted mainly by peritubular cells of the adult kidney, is the major factor regulating red blood cell (RBC) production [1]. Recombinant human EPO (rhEPO) has been approved for the treatment of anemia resulting from chronic renal failure, cancer chemotherapy, AIDS, etc. [2-4]. Administration of rhEPO as a potential therapeutic agent can reduce the necessity for blood transfusions and improve the patients' quality of life. Although rhEPO may be beneficial for the patients, the price of such a treatment prevents its use as a long-term intravenous treatment. Therefore, various strategies have been used to stimulate erythropoiesis. Many approaches to extend the half-life of EPO through genetic changes or chemical modification of native EPO have been considered in detail [5,6]. All these strategies have shown some effect on extending the half-life and enhancing the activities of rhEPO. Particularly, dimerization of 2 rhEPO peptides can significantly enhance the biological activity of the hormone; this is because the dimer has 2 high-affinity binding sites, resulting in better binding to the EPO receptor than is observed with the monomeric form of recombinant rhEPO [7-9]. Similarly, the longer half-life of novel erythropoietin stimulating protein (NESP), which was created by the introduction of 2 extra N-linked carbohydrate addition sites into the primary sequence of EPO, is likely to afford it a clinical advantage over rhEPO by allowing less frequent dosing in patients treated for anemia [10]. An EPO chimeric protein, constructed by fusing the carboxyl-terminal peptide of a human chorionic gonadotropin-β subunit bearing 4 O-linked oligosaccharide recognition sites with the coding sequence of human EPO cDNA, did not show altered secretion, receptor binding affinity, or in vitro bioactivity, but had significantly enhanced in vivo potency and half-life [11]. We also studied the production of rhEPO in mammalian cells and observed that hyperglycosylated rhEPO (HGEPO) and dHuEPO have higher erythropoietic activity than wild-type rhEPO, both in vitro and in vivo [12-14].

Transgenic (tg) animals are an attractive alternative to cell cultures for high-level, low-cost production of proteins. The mammary gland is the most reasonable organ for the production of recombinant proteins from transgenic organisms [15,16] and is suitable for synthesis of large amounts of protein that can be easily collected without causing harm to the animal [17]. Attempts have been made to obtain transgenic mice showing enhanced expression of the monomeric form of EPO [18-20]. We have also produced transgenic pigs expressing hEPO protein in the mammary gland, and showed that the purified hEPO had erythropoietic activity [21]. However, there has been no report on the generation of transgenic mice expressing the dHuEPO form. In the present study, we produced tg mice expressing dHuEPO, which was constructed by linking 2 human EPO molecules using a 2-amino acid peptide linker. dHuEPO tg mice developed excessive erythrocytosis that led to short lifespan, debility, and abnormal splenomegaly. Further, by microarray analysis, we have identified 2,672 genes associated with splenomegaly.

## Methods

### Construction of the dHuEPO gene

The N-terminal EPO domain of the human EPO dimer-encoding construct was amplified by polymerase chain reaction (PCR) with a plasmid containing the human EPO cDNA [12] using the primers EPO 1 (5'-TGG TCG ACA CCA TGG GGG TGC ACG AAT GTC CT-3'), which contains the *Sal*I site at the 5' end, and EPO 2 (5'-AGG ATA TCT CTG TCC CCT GTC CTG CAG GC-3'), which contains the Asp-Ile ligation site that was used to ligate 2 EPO molecules. With the exception of the stop codon, the complete EPO open-reading frame is present in this domain. The C-terminal EPO domain was constructed using the primers EPO 3 (5'-ATG ATA TCG CCC CAC CAC GCC TCA TC-3'), which contains the Asp-Ile ligation site and in which the signal sequence was removed, and EPO 4 (5'-TAC TCG AGT TCA TCT GTC CCC TGT CCT GCA-3'), which contains the *Sal*I site at the 3' end. This domain also contains the complete open-reading frame but not the signal sequence. The plasmid was constructed by ligation of the 6 nucleotide residues encoding the peptide linker fragment (Asp and Ile). The dimeric EPO molecule was constructed by the overlapping PCR method as previously reported [22]. The first PCR was performed using primers EPO 1-2 and 3-4. The resulting fragments were digested by *Xho*I/*Sal*I and ligated into the unique *Xho*I site of the expression vector pBC1 under the control of the goat β-casein promoter (designated as pBC1-dHuEPO). The direction of the ligated fragment was confirmed by restriction mapping using *Xho*I and *Sal*I. The sequence of the entire dHuEPO cDNA was verified by automated DNA sequencing performed as previously reported [22].

### Production and screening of transgenic mice

Tg mice were obtained by pronuclear microinjection of the dHuEPO cDNA driven by the goat β-casein promoter; microinjection was performed as previously described [23]. C57BL/6 N mice were used for the experiment. All mice were raised and maintained in the facilities of Macrogen Laboratories (Seoul, Korea). Potential tg mice were screened using genomic DNA samples obtained from tail biopsies. The PCR primers were as follows: EPO-1 F, 5'-CCC AGA ATC TAA GCG ATA TCT GGC-3' and EPO-1R, 5'-GCC CAG GAC TGG GAG GCC CAG AGG-3'. PCR was performed over 35 cycles (1 min at 94°C, 1 min at 56°C, and 1 min at 72°C). The predicted PCR product was 607 bp in length. The tg mice were bred by mating heterozygous male/female mice with wild-type females/males. The experiments were conducted according to the Guidelines for the Care and Use of Laboratory Animals, Hankyong National University.

### Blood analysis

Blood samples of the control and tg mice were obtained by heart puncture, collected in heparinized tubes, and placed on ice immediately. We examined 100 μL of blood on a HEMAVET 950 Automatic Cell Counter. Hematologic parameters examined included white blood cell (WBC) count, RBC count, and hematocrit (HCT).

### Reverse transcriptase-polymerase chain reaction (RT-PCR) analysis

Isolation of total RNA of frozen hearts, kidneys, livers, lungs, and spleens was performed using the TRIzol method (Invitrogen, Carlsbad, CA) according to the manufacturer's specifications. The final RNA sample was treated with DNase to prevent DNA contamination. For RT-PCR analysis, the reverse transcription reaction was performed with 8 μg of total RNA using SuperScript II Reverse Transcriptase and oligo(dT) primers according to the manufacturer's protocols. Two microliters of cDNA were used in each PCR reaction. The *dHuEPO *gene was detected using a forward primer (5'-ATG AGA ATA TCA CTG TCC CA-3') and a reverse primer (5'-GTG TCA GCA GTG ATT GTT CG-3'), which yielded 304- and 808-bp DNA fragments, respectively. The PCR conditions were 30 cycles of 30 s at 94°C, 30 s at 56°C, and 30 s at 72°C. Glyceraldehyde 3-phosphate dehydrogenase (GAPDH) was used for normalization of the dHuEPO expression, and the following GAPDH primer sequences were used for the normalization procedure: forward, 5'-ACC ACA GTC CAT GCC ATC AC-3' and reverse, 5'-TCC ACC ACC CTG TTG CTG TA-3'. The expected PCR fragment had a length of 452 bp. The PCR conditions were 26 cycles of 10 s at 98°C, 2 s at 55°C, and 20 s at 72°C.

### Splenectomy and histological analysis

Necropsy of the transgenic mice showed severe splenomegaly. The spleen was quickly removed for RNA preparation. Samples were kept in liquid nitrogen and stored individually at -80°C. The weight of the spleens of wild-type (wt) and tg mice was determined. Mice were necropsied, and the freshly dissected tissues from spleens were fixed in 10% formalin solution. Fixed specimens were embedded in paraffin and then cut into 4-μm-thick sections. The sections were stained with hematoxylin and eosin (H&E) according to standard protocols.

### Microarray analysis

Total RNA was extracted using the TRIzol reagent and purified using RNeasy columns (Qiagen, Valencia, CA) according to the manufacturer's protocol. After DNase digestion and clean-up procedures, the RNA samples were quantified, aliquoted, and stored at -80°C until use. For quality control, RNA purity and integrity were evaluated by performing denaturing gel electrophoresis and obtaining the optical density (OD) 260/280 ratio; these analyses were performed on an Agilent 2100 Bioanalyzer (Agilent Technologies, Palo Alto, CA).

#### Labeling and purification

Total RNA was amplified and purified using the Ambion Illumina RNA amplification kit (Ambion, Austin, TX) to yield biotinylated cRNA according to the manufacturer's instructions. Briefly, 550 ng total RNA was reverse-transcribed to cDNA using a T7 oligo(dT) primer. Second-strand cDNA was synthesized, in vitro transcribed, and labeled with biotin-NTP. After purification, the cRNA was quantified using the ND-1000 Spectrophotometer (NanoDrop, Wilmington, DE).

#### Hybridization and data export

We hybridized 750 ng of labeled cRNA samples to each Mouse-8 Expression Bead array for 16-18 h at 58°C according to the manufacturer's instructions (Illumina, Inc., San Diego, CA). The detection of the array signals was performed using Amersham Fluorolink streptavidin-Cy3 (GE Healthcare Bio-Sciences, Little Chalfont, UK) in accordance with the bead array manual. Arrays were scanned with an Illumina Bead Array Reader confocal scanner according to the manufacturer's instructions. Array data were exported and analysis was performed using Illumina BeadStudio v3.1.3 (Gene Expression Module v3.3.8).

#### Raw data preparation and statistical analysis

The quality of hybridization and overall chip performance was monitored by visual inspection of both internal quality controls and scanned raw data. The raw data were extracted using the software provided by the manufacturer (Illumina BeadStudio v3.1.3; Gene Expression Module v3.3.8). The array data were filtered by a detection *p*-value < 0.05 (similar to signal-to-noise ratio) in at least 50% of the samples (we applied a filtering criterion for data analysis; a higher signal value was required to obtain a detection *p*-value < 0.05). Selected gene signal values were transformed using a logarithm and normalized by the quantile method. The comparative analysis between the test and control groups was performed using the *t *test (adjusted Benjamini-Hochberg false discovery rate [FDR], 5% controlled) and fold-change values. Hierarchical cluster analysis was performed using complete linkage and Euclidean distance as measures of similarity. All data analyses and visualization of differentially expressed genes were conducted using ArrayAssist^® ^(Stratagene, La Jolla, CA) and R statistical language v. 2.4.0. Ontology-based analysis was performed using the Panther database [24].

### Quantitative real-time PCR (qRT-PCR)

To validate the microarray data, 20 genes from different categories, including platelet factor 4 (*Pf4*); Fc receptor, IgG, low affinity IV (*Fcgr4*); proteoglycan 2, bone marrow (*Prg2*); haptoglobin (*Hp*); elastase, neutrophil expressed (*Elane*); tribbles homolog 3 (*Trib3*); S100 calcium binding protein A9 (calgranulin B) (*S100a9*); pleckstrin 2 (*Plek2*); formyl peptide receptor 2 (*Fpr2*); histocompatibility 2, class II, locus Mb2 (*H2-DMb2*); chemokine (C-X-C motif) receptor 5 (*Cxcr5*); Fc receptor-like A (*Fcrla*); complement factor D (adipsin) (*Cfd*); C-type lectin domain family 4, member g (*Clec4g*); chemokine (C-C motif) receptor 6 (*Ccr6*); CD79B antigen (*Cd79b*); CD40 antigen (*Cd40*); CD6 antigen (*Cd6*); and inositol 1, 4, 5-triphosphate receptor 2 (*Itpr2*), were chosen for qRT-PCR analyses. Primer sequences and primer annealing temperatures are outlined in Table 1. Primers were designed using Primer3 software [25]. One microgram of total RNA was reverse transcribed to cDNA using oligo(dT) primers with the first-strand cDNA synthesis kit for RT-PCR (Invitrogen) according to the manufacturer's instructions. qRT-PCR was performed in a Rotor-Gene™ 6000 (Corbett Life Science, Mortlake, Australia). The reaction mixture consisted of 100 ng DNA, 4 nM of each primer, and 10 × qPCR Mastermix Plus (Toyobo, Osaka, Japan) in a total volume of 20 μL. A negative control containing all reagents minus DNA was included in each run. All reactions were performed with an initial denaturation for 10 min at 95°C followed by 40 cycles of 95°C for 10 s, the respective annealing temperature (Table 1) for 15 s, and 72°C for 20 s. The housekeeping gene β-actin was run in parallel. The threshold cycle number and reaction efficiency were determined using the Rotor-Gene 6000 series software version 6.1.93; the 2^-DDC^T method was used for relative quantitation. Absence of primers and the presence of the correct amplicon size for the specific SYBR green assays were verified by melting-curve analysis.

**Table 1:** The primer sets for genes used in qRT-PCR

	NCBI ACCESSION NUMBER	Forward	Tm	Reverse	Tm	size (bp)
**I**	NM_019932	CCGAAGAAAGCGATGGAGAT	57.3	TTCAGGGTGGCTATGAGCTG	59.3	139
	NM_144559	CCGTGGCATCAAATCACATT	59.4	CCTGAGGTTCCTTGCTCCAT	58.9	145
	NM_008920	CTTGCCTAGGGATGCAGAGA	59.4	ATCCTGACCTGAGACAGCTC	59.4	144
	NM_017370	GTGCCCGAGAAGAAAAACTTG	57.9	AGGTGTCCTCCTCCATGTCA	59.4	154
	NM_015779	GAGGCTGTGGATCTGGATTG	59.4	TCCTAGTTGGTCCTGCCCT	58.8	139
**II**	NM_144554	GGAGAACCTGGAAGATGCCT	59.4	GATCTTGCCAAAGAGCAGGA	57.3	142
	NM_175093	ACGACTCTGAGCCAGTCCT	58.8	CCACAAGTCGCTCTGAAGGT	59.4	132
	NM_009114	GACACCCTGAGCAAGAAGGA	59.4	AGCTCAGCTGATTGTCCTGG	59.4	139
	NM_013738	AGGGGCACAAGAGGAAAAAC	57.3	ACACAAGGGAACCACGAAGA	57.3	135
	NM_008039	GCCAGGACTTTCGTGAGAGA	59.4	CTTCATGGGGCCTTTAACTCA	57.9	142
**III**	NM_010388	TGGCTTTGTGGCTCATGTGG	59.4	CACCCCAAATTCACAGGGGA	59.4	148
	NM_007551	AACCGAGACCTTCCTGTTCC	59.1	TGTGCAGAGCGATCACAGTT	59	134
	NM_145141	GCAACCCAGATGTTACTGGC	59.1	CAGCTTCACTGGATTGGGTC	59.2	145
	NM_013459	ATCATGAACCGGACAACCTG	57.3	AACCACACCTTCGACTGCAT	57.3	150
	NM_029465	AAGCGCCAGAACAGCTCCT	58.8	ATCACCAGATGGGCACCCT	58.8	139
**IV**	NM_009835	GCTTTCCTGCATTGCTGCCT	59.4	TAAACCCGGGCACAGAGGAA	59.4	143
	NM_008339	ACAAGGATGACGGCAAGGC	58.8	ATTCCTGGCCTGGATGCTC	58.8	144
	NM_170702	ACAGCGGTCCATCTAGGGCA	61.4	CATGGGTGGCATTGGGTCTT	59.4	140
	NM_009852	CAACTTGGAACTGGCTGGC	58.8	AGGGGGTCCTGGACATTCA	58.8	148
	NM_019923	TGCCGTAATCAACACCAGCG	59.4	TCTGCAGGAAGGTGTGGGCT	61.4	136
**V**	β-actin	AGAGGGAAATCGTGCGTGAC	59.3	CAATAGTGATGACCTGGCCGT	59.8	138

(Biological process: I, Immunity and defense: II, Signal transduction; Molecular function: III, Defense/immunity protein: IV, Receptor; Housekeeping gene: V, β-actin)

## Results

### Production of dHuEPO tg mice

A total of 1,018 1-cell stage embryos were transferred to 52 recipient mice on day 1 of the estrous cycle (Table 2). Spontaneous abortion occurred in 23 recipients during mid-to-late pregnancy. Twenty-nine recipients (56%) successfully farrowed after embryo transfer. Thirty mice died soon after birth. The mortality rate of the tg mice was 39%, about 8-fold higher than that of the control wt mice. Forty-seven live mice were screened. Three tg mice were identified by transgene-specific PCR. Three positive transgenic founder lines, i.e., #3, #11, and #47, were obtained. One of these founders was female (#3) and the other 2 were male (#11, #47). All these founders produced offspring. PCR analysis of tail DNA samples revealed that only 1 (#11) of the 3 lines was positive. Two offspring (F1) (1 male, 1 female) of this founder (#11) were positive for the *dHuEPO *gene. Five mice were born from the F1 male, of which 3 (F2; 2 male, 1 female) were positive for the *dHuEPO *gene. The tg males (line #11), including F1 and F2 males, had a short life span. The average lifespan of male mice was 71-72 days. The remaining 2 female mice (F1, F2) developed a moribund condition. Moreover, the F1 and F2 female mice consistently aborted when they became pregnant. We performed autopsies on these 2 mice. Thus, we produced a total of 5 mice carrying the *dHuEPO *gene from founder #11. F1 transmission rate from founder was 100%. F2 transmission rate from F1 male was 60% (3/5).

**Table 2:** Embryo transfer and pregnancy rates of microinjected embryos

No. of 1-cell stage Transferred	No. of recipients farrowed/ no. of recipients (%)	No. of screened live offspring (%)	No. of founders (positive) (%)
1018	29/52 (56)	47 (4.6)	3 (6.4)

### Blood composition of dHuEPO tg mice

**Figure 1:**
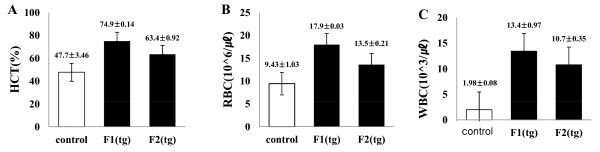
**Blood cell counts of dHuEPO transgenic mice**. Hematological parameters showed that tg mice had markedly increased RBC, WBC, and HCT values. All data are mean ± SD. A: HCT (%); B: RBC (× 10^6^/μL); C: WBC (× 10^3^/μL). HCT: hematocrit; RBC: red blood cell; WBC: white blood cell; F1 (tg): F1 female mice produced from founder #11; F2 (tg): F2 female mice produced from F1 founder male.

Mouse blood was collected by heart puncture. Blood analysis revealed that the RBC counts, WBC counts, and HCT values in the dHuEPO tg mice were remarkably higher than those in the wt mice (Figure 1). The total WBC count was higher in the blood of tg mice (wt, 1.93 ± 0.08 × 10^3^/μL vs. tg F1, 13.43 ± 0.97 × 10^3^/μL and tg F2, 10.77 ± 0.21 × 10^3^/μL). Similarly, the RBC count was higher in tg than in wt mice (wt, 9.43 ± 1.03 × 10^6^/μL vs. tg F1, 17.97 ± 0.03 × 10^6^/μL and tg F2, 13.57 ± 0.21 × 10^6^/μL). Notably, we also found that the HCT value was significantly higher in tg mice than in wt mice (wt, 47.75 ± 3.46% vs. tg F1, 74.9 ± 0.14% and tg F2, 63.45 ± 0.92%).

### Expression of dHuEPO mRNA in tg mice

**Figure 2:**
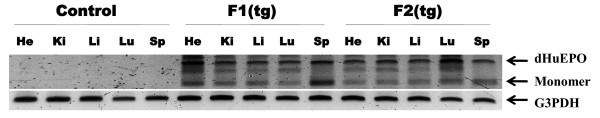
**Expression of dHuEPO mRNA in the tissues of the transgenic mice**. No PCR bands were detected in the control mice. The expected dimeric and monomeric PCR bands were found in the F1 (tg) and F2 (tg) mice. The mice tissues tested were heart (He), kidney (Ki), liver (Li), lung (Lu), and spleen (Sp).

We examined dHuEPO mRNA expression in various organs, and detected it using RT-PCR in the heart, kidney, liver, lung, and spleen (Figure 2). The 304- and 808-bp PCR amplicons were visualized on an agarose gel. The obtained PCR bands matched the predicted sizes. The expected PCR band was detected in most of the tissues examined indicating that tg mice expressed the *dHuEPO *gene in several tissues. We did not detect any PCR band in the wt mice.

### Tg mice showed distinct splenomegaly and an increased red pulp area

**Figure 3:**
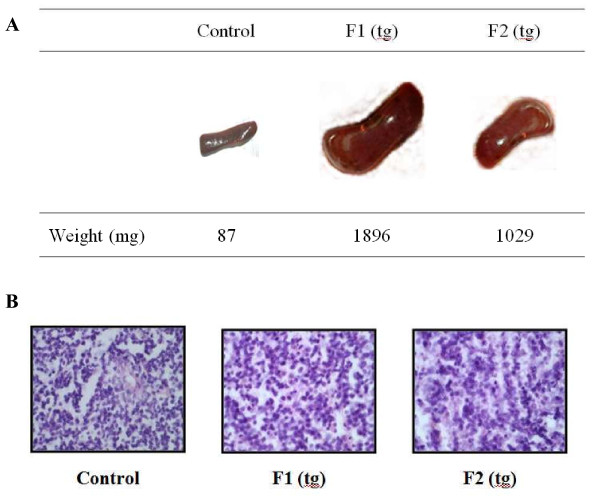
**Morphological changes and histological analysis of the spleen in splenomegaly**. **A**. Distinct splenomegaly-related changes were observed in the transgenic mice. The spleen weights of the transgenic mice were remarkably higher (21-fold increase in F1 and 11-fold increase in F2) than those of the control mice. **B**. Histological analysis of the spleen of the transgenic and control mice. Paraffin sections were stained with hematoxylin & eosin (original magnification, × 400).

The distinct differences in the spleens of tg mice are shown in Figure 3. Tg mice exhibited severe splenomegaly. The wet weights of spleens from the F1 and F2 tg mice were 21- and 11-fold higher than those of the spleens from wt mice (Figure 3A). When take the spleen, F1 female was 157 days in age and F2 female was 72 days in age. Thus, the spleen weight is difference between two mice. We observed H&E-stained sections of spleens of F1 tg, F2 tg, and wt mice (Figure 3B). The spleens of F1 tg and F2 tg mice were easily distinguishable from spleens of the wt mice. The tg spleens showed a greater red pulp area than did wt spleens.

### Microarray image and data analysis

**Figure 4:**
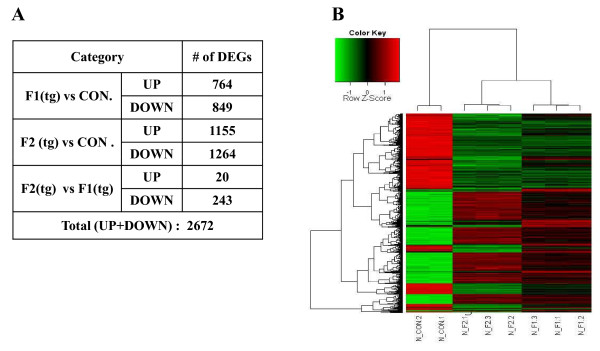
**Microarray image and data analysis**. **A**. Statistical significance of each correlation coefficient was determined using a corrected (Benjamini & Hochberg algorithm) *P *value (*P* < 0.05). **B**. Heat map and cluster analysis of gene expression measurements normalized to a single array showing genes in rows and samples in columns. Columns 1, 2: control mice; columns 3, 4, 5: F2 (tg); columns 6, 7, 8: F1 (tg); Con, control; tg, transgenic.

Using the microarray analysis tool, we assessed statistically significant variations in the expression levels of the 2,672 candidate genes (Figure 4A). In comparison with the expression in the wt control mice, 764 genes were upregulated and 849 were downregulated in spleens of F1 tg mice, 1,155 genes were upregulated and 1,164 were downregulated in F2 tg mice; and 20 genes were upregulated and 243 were downregulated in both F1 tg and F2 tg mice. Figure 4B shows the heat map (displaying differential expression levels) and hierarchical clustering of the chosen genes. The horizontal rows of the heat map represent genes, whereas the columns represent samples. Each pixel represents the expression of 1 gene in 1 experiment. Red represents upregulation, and green represents downregulation of gene expression, relative to wt levels. A greater portion of the heat map is green.

### Biological network classification of splenomegaly-associated genes

**Figure 5:**
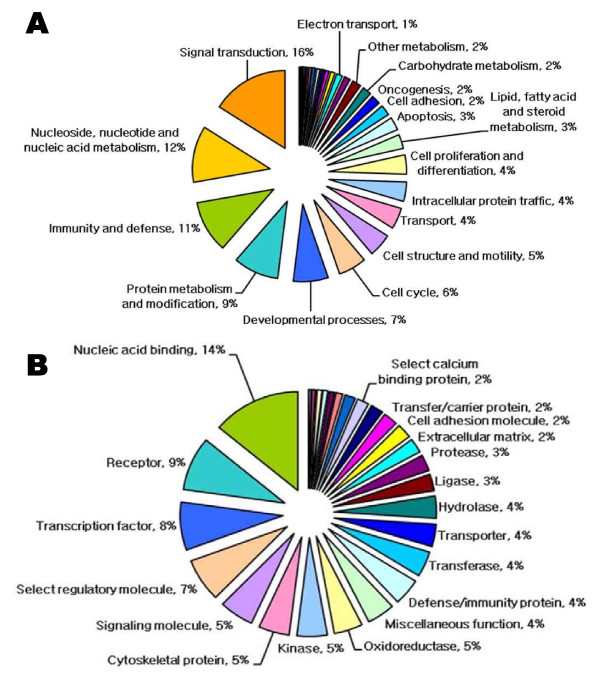
**Functional categorization of the genes showing significant transcriptional differences in the transgenic and control mice**. A total of 2,672 genes were categorized on the basis of Gene Ontology (GO) annotation, and the proportion of each category is displayed on the basis of biological process (A) and molecular function (B).

We used the Panther database to assign gene ontology (GO) categories to the 2,672 genes showing significant transcriptional differences between wt and tg mice. The categorization of splenomegaly-associated genes according to the molecular function and biological process is shown in Figure 5. Categorization based on biological processes revealed that the responsive genes were related to 29 biological processes, including signal transduction (16%), nucleoside, nucleotide, and nucleic acid metabolism (12%), immunity and defense (11%), protein metabolism and modification (9%), developmental processes (7%), cell cycle (6%), and cell structure and motility (5%) (Figure 5A). In the categorization based on molecular function, the genes were classified into 27 categories, as shown in Figure 5B. The most over-represented GO categories were concerned with nucleic acid binding (14%), receptors (9%), transcription factors (8%), select regulatory molecules (7%), and signaling molecules (5%). Additionally, we functionally categorized transcripts that showed a >5.0-fold increase in expression (data not shown). In this categorization, we obtained 12 categories for genes involved in biological processes, including signal transduction, immunity and defense, protein metabolism and modification, and nucleoside, nucleotide, and nucleic acid metabolism, and 13 categories for genes involved in molecular functions, including cytoskeletal proteins, defense/immunity proteins, and hydrolases. A total of 25 genes were classified, and we represented a range of changes observed in microarray studies (5.12- to 44.57-fold changes for upregulated genes; data not shown). A partial list of the transcripts that showed a <-5.0-fold change (decrease) in expression was also obtained (data not shown). Twenty-four of the gene categories in this list were associated with biological processes such as immunity and defense (34%), signal transduction (26%), developmental processes (8%), and protein metabolism and modification (5%), and 21 categories of genes were associated with molecular functions such as defense/immunity proteins (33%), receptors (32%), signaling molecules (14%), and kinases (12%). In this categorization, a total of 153 genes were classified that represented a range of changes observed in microarray studies (-5.01- to -81.38-fold changes for repressed genes; data not shown).

### Independent microarray validation using RT-PCR and qRT-PCR

**Figure 6:**
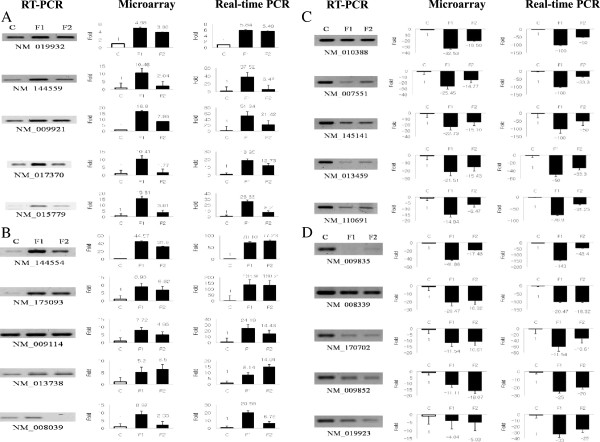
**Validation of independent microarray data using RT-PCR and qRT-PCR**. **A**. Upregulated genes in tg mice included 5 selected genes involved in defense and immunity. **B**. Upregulated genes involved in signal transduction in tg mice. **C**. Downregulated genes related to defense and immunity proteins in tg mice. **D**. Downregulated genes associated with receptors in tg mice. The relative expression levels were analyzed by RT-PCR (left) and real-time PCR (right). Columns and bars represent the means and standard error (n = 3) respectively. tg, transgenic.

Among all the cDNAs identified in the microarray study, we decided to narrow the search for candidate genes to 20 genes specific to the spleen (Table 3). These candidate genes were chosen according to the specific selection criterion of having a low or high impact on biological processes and molecular functions, e.g., genes associated with receptors, signal transduction, and immunity/defense. RT-PCR and qRT-PCR were conducted to validate the microarray results for the mRNA levels of the selected 20 clones (Figure 6). The 20 genes showed significantly different expression. The transcript abundance patterns of the control and tg mice were compared with the microarray data. All the genes analyzed showed the same expression patterns by qRT-PCR as noted in the microarray data. Figure 6 shows the expression levels of the 20 genes (10 genes showing upregulation and 10 showing downregulation). For example, in comparison with its expression in wt mice, the *Pf4 *gene (NM_019932) showed 5.4- to 5.8-fold upregulation in tg mice by qRT-PCR, consistent with the 3.8- to 4.9-fold higher expression seen in tg mice in the microarray data. Furthermore, the expression profiles of 10 genes, including *H2-DMb2 *(NM_010388), and 4 other genes with significant transcriptional changes, including [*Cxcr5 *(NM_007551), *Fcrla *(NM_145141), *Cfd *(NM_013459), and *Clec4g *(NM_110691)], were analyzed in the spleens of the control and tg mice. As expected, all these genes were downregulated in tg mice. Noticeably, the expression level of the *H2-DMb2 *(NM_010388) gene was 50-100-fold lower in tg mice than in wt mice.

**Table 3:** Genes differing in their expression levels between control and tg (F1 or F2) spleen

**No**.	NCBI Accession Number	Gene	F1/Con	F2/Con
Elevated in tg				
1	NM_019932	Platelet factor 4	4.98	3.86
2	NM_144559	Fc receptor, IgG, low affinity IV	10.46	2.04
3	NM_009921	Cathelicidin antimicrobial peptide	16.80	7.96
4	NM_017370	Haptoglobin	10.41	1.77
5	NM_015779	Elastase, neutrophil expressed	15.81	3.81
6	NM_144554	Tribbles homolog 3	44.57	31.60
7	NM_175093	Tribbles homolog 3 (Drosophila)	8.93	6.82
8	NM_009114	S100 calcium binding protein A9	7.72	4.95
9	NM_013738	Pleckstrin 2	5.30	6.50
10	NM_008039	Formyl peptide receptor 2	8.97	2.33
Reduced in tg				
11	NM_010388	Histocompatibility 2, class II, locus Mb2	-32.53	-19.50
12	NM_007551	Chemochine (C-X-C motif) receptor 5	-25.45	-14.77
13	NM_145141	Fc receptor-like A	-22.73	-15.10
14	NM_013459	Complement factor D (adipsin)	-21.51	-15.43
15	XM_110691	C-type lectin domain family 4, member g	-14.94	-6.47
16	NM_009835	Chemokine (C-C motif) receptor 6	-41.86	-17.43
17	NM_008339	CD79B antigen	-23.98	-20.22
18	NM_170702	CD40 antigen	-11.54	-10.61
19	NM_009852	CD6 antigen	-11.11	-16.07
20	NM_019923	Inositol 1,4,5-triphosphate receptor 2	-4.04	-5.03

Gene names correspond to their Unigene identification where possible, and otherwise their Affymetrix identifier. Genes are displayed from the greatest to least fold-change and the specified tg/control expression ratio is indicated

## Discussion

The dimer of 2 human EPO molecules linked by peptide linkers shows higher erythropoietic activity than the monomeric molecule, and this enhanced activity was observed both in vitro in primary human erythroid progenitors and in vivo in normal mice [7,9]. On the basis of these results, an expression vector producing dHuEPO protein was constructed to utilize the fusion system of 2 human EPO molecules linked by a peptide linker of 2 amino acids (Asp-Ile). Several medical proteins have been successfully produced using goat promoter systems. Using the goat β-casein promoter, therapeutic proteins have been expressed at high levels in the milk of tg mice [26]. We designed the dHuPEO gene by recombinant DNA-mediated fusion of EPO coding regions linked by the Asp-Ile peptide and analyzed the physiological characteristics of the dHuEPO transgenic mice. The dHuEPO tg mice exhibited splenomegaly and abnormal blood composition. The inserted *dHuEPO *gene was detected in all mouse tissues tested. Most of the tg mice tended to show abnormal symptoms, e.g., short life span. We identified 2,672 candidate genes in the spleen by microarray analysis; more genes were downregulated than upregulated in tg mice showing splenomegaly.

We found that the spontaneous abortion rate was high in the tg mice. Twenty-nine recipients (56%) successfully farrowed after dHuEPO tg embryo transfer. Song et al. [27] reported that lanosterol 14α-demethylase (LDM) is selectively expressed in preimplantation embryos and the uterine subluminal stroma surrounding the implanting blastocyst on day 5 of pregnancy. A high level of LDM expression is also observed in the uterus deciduas on day 6-8 of pregnancy, indicating that LDM is closely related to mouse embryo implantation. Therefore, dHuEPO embryos maybe have perturbation in sterol biosynthesis and metabolism during the peri-implantation period, leading to a high rate of spontaneous abortion or death. We observed that tg males, including F1 and F2 individuals, had a short life span. Several groups have reported premature mortality in polycythemic mice [18,28-30]. In contrast, other groups did not observe shortened life span in a series of human EPO tg mouse lines with elevated mean HCTs ranging from 48% to 80% [31-33]. In the present study, the F1 and F2 tg mice tested had HCT values of 74% and 63%, respectively. The HCT values of the tg mice were markedly higher than that of the controls (47%). Madan et al. [34] suggested that different mouse strains may differ in their ability to tolerate the rheological and hemodynamic effects of increased blood viscosity due to an elevated HCT. Kim et al. [35] reported that the life span of hEPO tg mice may be affected, and these animals may die of microcytic anemia and acute leukemia. The tg females (F1/F2) experienced spontaneous abortion during mid-to-late pregnancy. Thus, we were unable to obtain dHuEPO protein in their milk. Toth et al. [36] reported that EPO-receptor (EPO-R) expression in the villous trophoblast of the abortion tissue in tg mice was significantly higher than the normal EPO-R levels in humans. Although there is no experimental evidence to support the direct cause of these abortions, it is possible that the expression of EPO-R is upregulated in the placenta of the abortion tissue. EPO levels in the amniotic fluid correlate well with the EPO levels in the cord plasma and are significantly higher in pregnancies complicated by hypertension than in normal pregnancies [37].

The ectopic expression of a transgene can be influenced by the site of integration, the absence of specific regulatory elements in the promoter, and the presence of negative regulatory elements [38,39]. Ectopic expression of the *EPO *gene has been shown to cause harmful effects on the survival, health, and growth of some tissues in tg mice [35,40]. In our study, tg mice expressed dHuEPO mRNA in a variety of tissues, including the heart, kidney, liver, lung, and spleen. Thus, it is possible that ectopic expression of dHuEPO caused multiple organ failure in tg mice. F1 and F2 tg mice showed splenomegaly, and the weight of the spleen increased by 21- and 11-fold, respectively. A previous study reported that enhanced erythropoiesis occurred in tg spleens, accompanied by an up to 5-fold increase in weight [20]. In studies of inpatients with splenomegaly, hematological diseases were positively associated with lymphadenopathy, massive splenomegaly, and cytosis (erythrocytosis, leukocytosis, and thrombocytosis) [41,42]. O'Reilly et al. [43] reported that 84% of the cases with progressive splenic enlargement were associated with hematological disease, predominantly malignancy. In the present study, the RBC counts were higher in the tg mice than in the controls, which might have caused an increase in the spleen weight of the tg mice carrying the dHuEPO gene. The tg spleens showed a higher red pulp area than did wt spleens. Histological analysis revealed extramedullary erythropoiesis in the spleen, and erythropoietic activity was visualized using the monoclonal antibody ER-HR3 [20]. Our data indicated a similar incidence of massive splenic erythropoiesis in tg mice. Both extramedullary erythropoiesis and splenomegaly could cause classic complications in human patients suffering from polycythemia vera [44]. In the present study, we found that the HCT values in tg mice were higher than those in the controls (wt, 47% vs. tg F1, 74% and tg F2, 63%). Similar to these observations, the HCT values increased from 0.41 to 0.89 in tg mice. HCT levels of splenectomized tg mice were reduced by about 30% from 0.89 to 0.62 [20]. Most of the EPO tg mice showed severe nerve fiber degeneration of the sciatic nerve, a decreased number of neuromuscular junctions, and degeneration of skeletal muscle fibers. Thus, chronically increased EPO levels induced excessive erythrocytosis and led to multiple organ degeneration, thereby providing an explanation for the reduced life expectancy [19]. Erythrocyte aging of EPO tg mice was observed to be accelerated, which, together with an increased number and activity of macrophages, resulted in enhanced erythrocyte clearance [18]. These results indicate that extramedullary erythropoiesis can cause splenomegaly in tg mice.

A number of studies have employed microarray technology to characterize gene expression profiles [45-47]. Accordingly, this study was designed to provide data on the changes in the gene expression profile in the spleen of dHuEPO tg mice. Our results showed that a total of 2,672 genes were differentially expressed in the spleen of tg mice, in comparison to their expression in the controls. Furthermore, the expression of 153 of these genes (< 5-fold change, *P <*0.05) was repressed, and the expression of 25 genes (>5-fold change, *P <*0.05) was promoted. The tg spleens had more downregulated genes than the controls, including genes for defense/immunity proteins and receptor-related genes; this suggests that the spleen is a major site for immunological elimination. Commonly used techniques for validation of microarray data include RT-PCR, qRT-PCR, northern blot, ribonuclease protection assay, and in situ hybridization or immunohistochemistry [48]. We used RT-PCR and qRT-PCR to validate our microarray data. Although the standard deviations in the expression levels of validated genes tended to be different, they matched the microarray patterns for the most part. These changes in gene expression profiles may provide a molecular framework to explain the complex differences in the splenic phenotypes of dHuEPO tg mice. Moreover, these results provide a global picture of gene expression differences in splenomegaly.

## Conclusions

We generated dHuEPO tg mice using the goat β-casein promoter system, but this system had a negative effect on survival. Moreover, we were unable to obtain dHuEPO protein from the milk of these mice. Tg mice caused excessive erythrocytosis that led to abnormal blood composition, short lifespan, and abnormal splenomegaly. We have identified 2,672 genes associated with splenomegaly by microarray analysis in these mice. Thus, further studie is required to define these symptoms (excessive erythrocytosis, short lifespan and excessive splenomegaly) in dHuEPO tg mice.

## Competing interests

The authors declare that they have no competing interests.

## Authors' contributions

SJY, PN, CWP, and TN performed the experiments. JJP, BWS, and MHK drafted the manuscript. SYH, JTY, and KSM designed supervised the experimental work, and revised the manuscript. All authors read and approved the final manuscript.
